# Development and validation of a nomogram to predict the risk of vancomycin-related acute kidney injury in critical care patients

**DOI:** 10.3389/fphar.2024.1389140

**Published:** 2024-08-28

**Authors:** Peng Bao, Yuzhen Sun, Peng Qiu, Xiaohui Li

**Affiliations:** ^1^ Fuwai Central China Cardiovascular Hospital, Zhengzhou University, Zhengzhou, China; ^2^ Department of Rehabilitation, First Affiliated Hospital of Wenzhou Medical University, Wenzhou Medical University, Wenzhou, China

**Keywords:** vancomycin, acute kidney injury, nephrotoxicity, nomogram, critical care patients

## Abstract

**Background:**

Vancomycin-associated acute kidney injury (AKI) leads to underestimated morbidity in the intensive care unit (ICU). It is significantly important to predict its occurrence in advance. However, risk factors and nomograms to predict this AKI are limited.

**Methods:**

This was a retrospective analysis of two databases. A total of 1,959 patients diagnosed with AKI and treated with vancomycin were enrolled from the Medical Information Mart for Intensive Care IV (MIMIC-IV) database. According to the 7:3 ratio, the training set (n = 1,372) and the internal validation set (n = 587) were randomly allocated. The external validation set included 211 patients from the eICU Collaborative Research Database (eICU). Next, to screen potential variables, the least absolute shrinkage and selection operator (LASSO) regression was utilized. Subsequently, the nomogram was developed by the variables of the selected results in the multivariable logistic regression. Finally, discrimination, calibration, and clinical utility were evaluated to validate the nomogram.

**Results:**

The constructed nomogram showed fine discrimination in the training set (area under the receiver operator characteristic curve [AUC] = 0.791; 95% confidence interval [CI]: 0.758–0.823), internal validation set (AUC = 0.793; 95% CI: 0.742–0.844), and external validation set (AUC = 0.755; 95% CI: 0.663–0.847). Moreover, it also well demonstrated calibration and clinical utility. The significant improvement (*P* < 0.001) in net reclassification improvement (NRI) and integrated differentiation improvement (IDI) confirmed that the predictive model outperformed others.

**Conclusion:**

This established nomogram indicated promising performance in determining individual AKI risk of vancomycin-treated critical care patients, which will be beneficial in making clinical decisions.

## Introduction

Vancomycin, a potent glycopeptide high-grade antibiotic, has been used for more than six decades to combat infections caused by Gram-positive, beta-lactam-resistant bacteria (Rybak et al., 2020a). However, its clinical use is complicated by several issues. The therapeutic window of vancomycin is narrow, and patient reactions differ significantly from one another. In addition, nephrotoxicity is its most severe adverse effect, with the incidence of vancomycin-associated acute kidney injury (AKI) reported between 16.1% (Xu et al., 2020) and 72% (Li et al., 2022). When AKI occurs, vancomycin treatment must be altered or discontinued, which can lead to longer hospital stays and higher mortality rates (Prendecki et al., 2016). Early identification and prevention of vancomycin-related kidney injury are crucial for improving the prognosis of severe AKI patients (van Hal et al., 2013; Jeffres, 2017). However, accurate prediction is still difficult.

Fortunately, the clinical prediction model may be beneficial to solve this issue. Determining the risk factors related to AKI and constructing a clinical prediction model can aid in predicting the incidence of vancomycin-associated AKI (Collins et al., 2015; Park, 2018). Extensive studies have been conducted to identify risk factors associated with vancomycin-induced acute renal insufficiency (Kim et al., 2022b; Yousif and Awdishu, 2023). By incorporating a variety of risk factors, predictive models can help clinicians assess the risk of AKI following vancomycin administration. However, existing predictive models for vancomycin-associated AKI are limited.

The model proposed by Zheng does not meet the requirement of 10 events per variable (10 EPV) because too many candidate indicators were included in a limited sample size (Guo et al., 2021; Riley et al., 2020). Moreover, the use of logistic univariate analysis fails to overcome the collinearity problem of the screened variables (Xu et al., 2020). The model proposed by Imai, presented as a decision tree (Miyai et al., 2020), lacks vancomycin-related indicators. In addition, the variables based on the selection of logistic multivariate analysis and variables utilized in the decision tree are not completely consistent. The model proposed by Kim et al. (2022a), derived from a very small cohort of only 104 patients and diagnosed by the Acute Kidney Injury Network (AKIN) standards (Mehta et al., 2007) rather than the more extensively utilized Kidney Disease Improving Global Outcome (KDIGO) criteria (Kellum et al., 2013), potentially leads to interpretative heterogeneity (Kim et al., 2022a). There is a need for a novel and more accurate model to predict vancomycin-associated AKI in the ICU patient population.

To address the research gap, this study is designed to establish and validate a prediction model that could precisely predict the occurrence of vancomycin-related AKI.

## Methods

### Data source

The data utilized in this study were derived from the Medical Information Mart for Intensive Care (MIMIC) IV and eICU Collaborative Research Database (eICU). MIMIC-IV, a substantial public database funded by multiple institutions, was established in 2003, sponsored by the National Institutes of Health. MIMIC-IV 2.2 is the latest version, updated in January 2023, and encompasses detailed patient data from ICU admissions in the emergency department spanning 2008–2019 (Johnson et al., 2023). As all personal information has been removed from the database, this study did not require informed consent. We completed the necessary online courses and an examination to obtain authorization for accessing this database (Record ID: 60692864). In addition, the eICU database is available under the Health Insurance Portability and Accountability Act (HIPAA) safe harbor provision (Certification No. 1031219-2); therefore, ethics approval was not required.

### Population selection criteria

Inclusion criteria: This study focused on patients who were admitted to the intensive care unit (ICU) for the first time and received vancomycin treatment during their ICU stay. Eligible patients had recorded the start and stop times of vancomycin administration and at least one measured vancomycin trough concentration.

Exclusion criteria: 1) Occurrence of AKI prior to vancomycin use or more than 72 h after discontinuation; 2) the existence of chronic kidney disease before vancomycin administration; 3) continuous renal replacement therapy (CRRT) was used during vancomycin treatment; 4) vancomycin administration for less than 48 h; 5) an ICU stay shorter than 48 h; 6) patients under 16 years of age; and 7) excessive missing patient information (Supplementary Figure S1).

### Clinical variables and outcome definition

This study involved the extraction of various variables during the first 24 h of ICU admission, which included demographics, admission status, vancomycin use status, comorbidities, the source of infection, infectious pathogen, concomitant nephrotoxic agent, vital signs, laboratory tests, and outcomes.

The demographics and admission status comprised age, weight, gender, sequential organ failure assessment (SOFA) score, Glasgow Coma Scale (GCS), and the status of ventilator and vasopressor use. Vancomycin-related indicators include vancomycin trough concentration and vancomycin duration. Comorbidities contained preexisting sepsis, congestive heart failure, coronary heart disease, chronic obstructive pulmonary disease (COPD), diabetes, hypertension, renal disease, pulmonary disease, acquired immunodeficiency syndrome (AIDS), malignancy, hepatic disease, and the Charlson Comorbidity Index (CCI). The source of infection included the lung, urinary tract, abdomen, catheter, skin, and cranial cavity. The infectious pathogen included methicillin-resistant *Staphylococcus aureus (MRSA)*, *Streptococcus*, *Staphylococcus*, *Escherichia*, *Enterococcus*, and others/unclear. Whether the co-infection pathogens include Gram-negative bacteria was documented. Concomitant nephrotoxic agents included angiotensin-converting enzyme inhibitors/angiotensin II receptor blockers (ACEIs/ARBs), non-steroidal anti-inflammatory drugs (NSAIDs), acyclovir, aminoglycosides, contrast agents, amphotericin B, cyclosporine, loop diuretics, piperacillin–tazobactam, tacrolimus, and vasopressors. The number of concomitant nephrotoxic agents was also recorded. Vital signs included temperature, heart rate (HR), respiratory rate (RR), and mean blood pressure (MBP). The white blood cell (WBC), hemoglobin, platelet, blood urea nitrogen (BUN), sodium, potassium, chloride, bicarbonate, creatine, and glucose levels can be found in the laboratory test section. Additional outcomes encompassed the length of stay (LOS) in the ICU and hospital, as well as hospital mortality.

According to the KDIGO criteria (Kellum et al., 2013), AKI was diagnosed with daily serum creatinine (SCr) concentrations. Thus, AKI was identified by an increase in SCr levels by more than 1.5 times compared to the baseline value or an elevation of ≥0.3 mg/dL within 48 h. The lack of these KDIGO SCr criteria did not denote the presence of AKI.

### Statistical analysis

We used multiple imputations to impute missing values (White et al., 2011). Statistical analyses were conducted utilizing R version 4.3.2 and SPSS 25.0 software. Continuous data were shown as the mean with standard deviation (SD) or median with interquartile range (IQR), while categorical data were demonstrated as percentages (%). The χ^2^ test or Fisher’s exact test and the Mann–Whitney U test were carried out for comparing categorical variables and continuous variables, respectively. A 7:3 ratio was used to divide the patients into a training set and a validation set. Spearman’s correlation analysis was utilized to evaluate the presence of correlation across all the included variables (Dong et al., 2022). The least absolute shrinkage and selection operator (LASSO) regression was carried out among the included variables using the outcome of the AKI function as the dependent variable. Meanwhile, the LASSO regression recalculates variable coefficients in order to avoid overfitting and overcome significant collinearity concerns (Lei et al., 2023; Sauerbrei et al., 2007). The initial multivariable logistic regression model, including all LASSO-selected predictors, was developed to predict AKI and create a nomogram. Significant predictors (*p* < 0.05) were selected using a backward stepwise approach, with the choice of final variables chosen on the basis of the minimum Akaike information criterion (AIC).

The discrimination between AKI and non-AKI was validated utilizing the area under the curve (AUC), with AUC >0.7 indicating acceptable discrimination. Calibration curves evaluated the consistency between the predicted and actual results, with a *p*-value >0.05 in the Hosmer–Lemeshow (HL) test signifying good calibration. In addition, Brier scores were also utilized to demonstrate the goodness of fit as another indicator of calibration plots. Both discrimination and calibration tests were assessed utilizing bootstrapping methods (1,000 resamples). In addition, decision curve analysis (DCA) assessed the clinical utility of the model. Finally, model comparisons were made using net classification improvement (NRI) and integrated discrimination improvement (IDI) (Wu et al., 2021).

## Results

### Patient characteristics

In our retrospective analysis of ICU clinical records, 5,425 patients fulfilled the inclusion criteria, with 1,959 patients ultimately included in the study. The incidence of vancomycin-induced AKI among patients was 84.4% (1,654/1,959). The differences in the baselines of patients between the AKI and non-AKI groups are indicated (Table 1). Patients in the AKI group were older, and had higher body weight, lower GCS, and higher SOFA than those in the non-AKI group. They also exhibited an elevated use of ventilators and vasopressors. In addition, compared with patients of the non-AKI group, AKI patients tend to have a higher vancomycin trough concentration and longer vancomycin duration time. The higher incidence of comorbidities, including sepsis, congestive heart failure, hypertension, COPD, coronary heart disease, pulmonary diseases, hepatic diseases, and a higher CCI, was more prevalent in the AKI group. These patients were more likely to suffer from pulmonary, abdominal, and catheter-associated infections, as well as MRSA invasion, but had lower rates of *Streptococcus* infection. The use of loop diuretics, vasopressors, and multiple nephrotoxic agents was higher in the AKI group. While respiratory rates were elevated in AKI patients, other vital signs showed no significant differences. Compared to the non-AKI group [6.56%, 4.1 (2.8, 6.3) days, and 11.2 (7.3, 18.3) days], hospital mortality and the LOS in both the ICU and hospital were markedly higher than those in the AKI group [20.4%, 7.72 (4.7, 13.4) days, and 15.1 (9.8, 23.5) days] (*p* < 0.001). The differences in patient characteristics between the training and validation sets were not significant (Supplementary Table S1).

**TABLE 1:** Characteristics at the baseline between the non-AKI and AKI groups.

	All	Non-AKI	AKI	*p*-value
	N = 1,959	N = 305	N = 1,654	
Age (year)	62.9 (51.0, 74.3)	58.4 (46.1, 69.7)	63.7 (52.2, 75.1)	<0.001
Gender (male)	1,093 (55.8%)	163 (53.4%)	930 (56.2%)	0.403
Weight (kg)	79.4 (65.9, 97)	71.3 (60.1, 86.0)	80 (67.5, 99.1)	<0.001
GCS	11 (7, 14)	13 (9, 15)	10 (7, 14)	<0.001
SOFA	7 (4, 10)	4.91 (3.02)	7 (5, 11)	<0.001
Ventilator	1,484 (75.8%)	178 (58.4%)	1,306 (79.0%)	<0.001
Vasopressor	1,215 (62.0%)	124 (40.7%)	1,091 (66.0%)	<0.001
Vancomycin trough concentration (mg/L)	15 (9.9, 20.5)	11.6 (7.7, 17.1)	15.7 (10.6, 21.1)	<0.001
Vancomycin duration (h)	127 (75, 227)	91 (70, 168.5)	136 (77, 242.3)	<0.001
Comorbidities				
CCI	5 (3, 6)	4.18 (2.76)	5 (3, 7)	<0.001
Sepsis	1,798 (91.8%)	256 (83.9%)	1,542 (93.2%)	<0.001
Congestive heart failure	471 (24.0%)	36 (11.8%)	435 (26.3%)	<0.001
Hypertension	941 (48.0%)	121 (39.7%)	820 (49.6%)	0.002
COPD	352 (18.0%)	37 (12.1%)	315 (19.0%)	0.005
Diabetes	452 (23.1%)	59 (19.3%)	393 (23.8%)	0.108
Coronary heart disease	345 (17.6%)	34 (11.1%)	311 (18.8%)	0.002
Pulmonary disease	1,665 (85.0%)	222 (72.8%)	1,443 (87.2%)	<0.001
AIDS	24 (1.23%)	6 (1.97%)	18 (1.09%)	0.25
Malignancy	284 (14.5%)	42 (13.8%)	242 (14.6%)	0.761
Hepatic disease	350 (17.9%)	30 (9.84%)	320 (19.3%)	<0.001
Source of infection				
Pulmonary infection	1,233 (62.9%)	169 (55.4%)	1,064 (64.3%)	0.004
Genitourinary infection	352 (18.0%)	43 (14.1%)	309 (18.7%)	0.067
Abdominal infection	384 (19.6%)	41 (13.4%)	343 (20.7%)	0.004
Catheter-associated infection	285 (14.5%)	27 (8.85%)	258 (15.6%)	0.003
Skin infection	208 (10.6%)	42 (13.8%)	166 (10.0%)	0.065
Intracranial infection	98 (5.00%)	20 (6.56%)	78 (4.72%)	0.225
Infectious pathogen				
MRSA	164 (8.37%)	16 (5.25%)	148 (8.95%)	0.042
*Staphylococcus*	671 (34.3%)	98 (32.1%)	573 (34.6%)	0.433
*Streptococcus*	174 (8.88%)	43 (14.1%)	131 (7.92%)	0.001
*Escherichia*	166 (8.47%)	28 (9.18%)	138 (8.34%)	0.711
*Enterococcus*	160 (8.17%)	19 (6.23%)	141 (8.52%)	0.218
Others/unclear	1,026 (52.4%)	161 (52.8%)	865 (52.3%)	0.924
Co-infection with Gram-negative bacilli	961 (49.1%)	152 (49.8%)	809 (48.9%)	0.815
Concomitant nephrotoxic agent				
ACEI/ARBs	77 (3.93%)	8 (2.62%)	69 (4.17%)	0.263
Acyclovir	86 (4.39%)	13 (4.26%)	73 (4.41%)	1
Aminoglycosides	45 (2.30%)	7 (2.30%)	38 (2.30%)	1
Amphotericin B	8 (0.41%)	2 (0.66%)	6 (0.36%)	0.361
Contrast agents	155 (7.91%)	15 (4.92%)	140 (8.46%)	<0.001
Cyclosporine	2 (0.10%)	0 (0.00%)	2 (0.12%)	1
Loop diuretics	526 (26.9%)	44 (14.4%)	482 (29.1%)	<0.001
NSAIDs	795 (40.6%)	121 (39.7%)	674 (40.7%)	0.773
Piperacillin tazobactam	321 (16.4%)	38 (12.5%)	283 (17.1%)	0.053
Tacrolimus	10 (0.51%)	2 (0.66%)	8 (0.48%)	0.66
No. of nephrotoxic drugs	0 (0, 2)	0 (0, 2)	0 (0, 2)	0.001
Vital signs				
Heart rate (beats/min)	96 (82, 111)	98 (82, 111)	96 (82, 111)	0.86
MBP (mmHg)	81 (70, 93)	81 (71, 94)	81 (70, 93)	0.875
Temperature (◦C)	36.9 (36.5, 37.4)	37.1 (36.6, 37.4)	36.9 (36.5, 37.4)	0.113
Respiratory rate (breaths/min)	20 (17, 25)	20 (16, 24)	21 (17, 25)	0.02
Laboratory tests				
WBC (K/uL)	12.9 (8.8, 18.3)	12.6 (8.1, 17.3)	12.9 (8.9, 18.5)	0.879
Hemoglobin (g/dl)	11.0 (9.4, 12.9)	11.2 (9.2, 12.7)	11 (9.4, 13)	0.082
Sodium (mmol/L)	138 (135, 141)	137 (135, 140)	138 (135, 141)	0.132
Potassium (mmol/L)	4.1 (3.7, 4.6)	4 (3.5, 4.4)	4.1 (3.7, 4.6)	0.004
Chloride (mmol/L)	103 (99, 108)	103 (99, 106)	103 (99, 108)	0.15
Bicarbonate (mmol/L)	23 (20, 26)	23 (20, 26)	22.7 (20, 26)	0.197
Creatinine (mg/dl)	0.9 (0.7, 1.2)	0.8 (0.6, 1.2)	0.9 (0.7, 1.3)	0.648
BUN (mg/dL)	18 (13, 29)	15 (11, 24)	19 (13, 30)	0.025
Glucose (mg/dl)	132 (108, 174)	127 (107, 161.5)	133 (108, 177)	0.331
Outcomes				
LOS ICU (days)	7.0 (4.1, 12.8)	4.1 (2.8, 6.3)	7.72 (4.7, 13.4)	<0.001
LOS hospital (days)	14.5 (9.1, 22.8)	11.2 (7.3, 18.3)	15.1 (9.8, 23.5)	<0.001
Hospital mortality	357 (18.2%)	20 (6.56%)	337 (20.4%)	<0.001

Characteristics are summarized as the median (Q1, Q3) or frequency (%). AKI, acute kidney injury; GCS, Glasgow Coma Scale; SOFA, sequential organ failure assessment; CCI, Charlson Comorbidity Index; COPD, chronic obstructive pulmonary disease; AIDS, acquired immunodeficiency syndrome; MRSA, methicillin-resistant *Staphylococcus aureus*; ACEIs/ARBs, angiotensin-converting enzyme inhibitors/angiotensin receptor blockers; NSAIDs, non-steroidal anti-inflammatory drugs; MBP, mean blood pressure; WBC, white blood cell; BUN, blood urea nitrogen; LOS, length of stay.

### Variable selection and establishment of a nomogram

**FIGURE 1:**
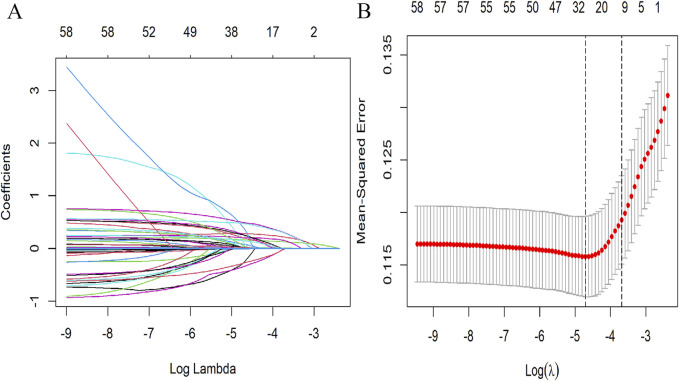
Selection of demographic and clinical features utilizing LASSO regression. **(A)** Tuning parameter (λ) selection using LASSO penalized regression. **(B)** LASSO coefficient profiles of the variables. In this plot, each colored line represents the coefficient of a specific variable, illustrating the influence of each variable within the LASSO regression. LASSO, least absolute shrinkage and selection operator.

Spearman’s correlation analysis was utilized to evaluate the collinearity among the screened variables before we conducted the regression analysis (Supplementary Figure S2). The LASSO regression was applied to 61 candidate variables, resulting in the selection of 10 variables (Figure 1; Supplementary Figure S3). The multivariable logistic analysis of the eight variables finally included is detailed in Table 2. The final model incorporated eight predictors: age (odds ratio [OR]: 1.02; 95% CI: 1.01–1.03), weight (OR: 1.02; 95% CI: 1.01–1.03), vancomycin trough concentration (OR: 1.02; 95% CI: 1.00–1.04), vancomycin duration (OR: 1.00; 95% CI: 1.00–1.00), SOFA score (OR: 1.42; 95% CI: 1.12–1.25), congestive heart failure (OR: 2.08; 95% CI: 1.27–3.39), pulmonary diseases (OR: 1.53; 95% CI: 1.04–2.26), and loop diuretics (OR: 2.06; 95% CI: 1.3–3.26) (Table 2).

**TABLE 2:** Multivariate logistic regression model of AKI in the training set.

	B	SE	OR	CI	Z	P
Age	0.015	0.005	1.02	1.01–1.03	3.156	0.002
Weight	0.021	0.004	1.02	1.01–1.03	5.136	<0.001
Vancomycin trough concentration	0.022	0.01	1.02	1.00–1.04	2.202	0.028
Vancomycin duration	0.002	0.001	1.00	1.00–1.00	3.179	0.001
SOFA score	0.165	0.028	1.18	1.12–1.25	5.831	<0.001
Congestive heart failure	0.73	0.251	2.08	1.27–3.39	2.91	0.004
Pulmonary disease	0.428	0.198	1.53	1.04–2.26	2.16	0.031
Loop diuretics	0.723	0.234	2.06	1.3–3.26	3.092	0.002

B, regression coefficient; SE, standard error; OR, odds ratio; CI, confidence interval; SOFA: sequential organ failure assessment.

**FIGURE 2:**
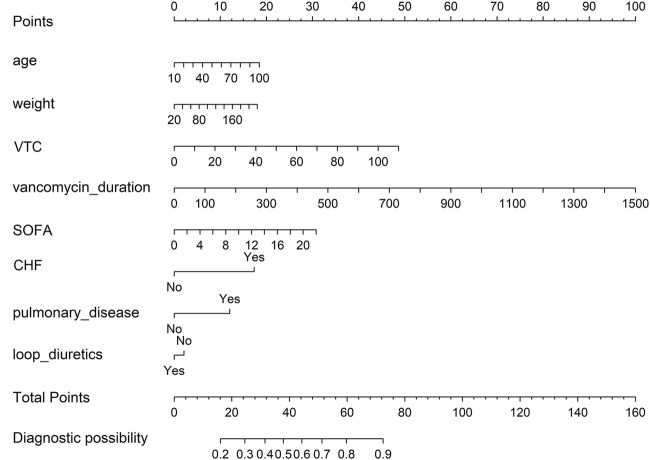
Nomogram to detect the risk of vancomycin-associated AKI, based on the LASSO and logistic regression analyses. To utilize this nomogram, a vertical line is drawn from each variable to the “points” axis to determine individual scores. These scores are aggregated to find the total on the “total points” axis. Then, a vertical line is drawn from this total point straight down to the “probability of AKI” axis to estimate the AKI risk. AKI, acute kidney injury; VTC, vancomycin trough concentration; SOFA, sequential organ failure assessment; CHF, congestive heart failure.

We constructed a nomogram to evaluate the probability of vancomycin-related AKI in ICU patients (Figure 2). The probability of AKI was evaluated utilizing this nomogram by calculating the total score (Supplementary Figure S4).

### Performance of the nomogram

**FIGURE 3:**
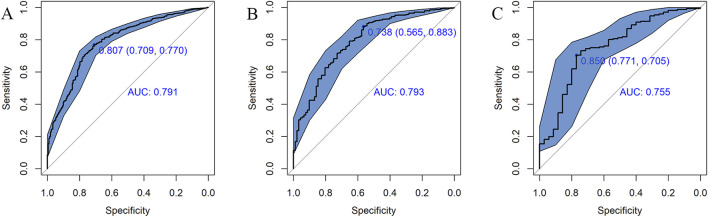
ROC curves for the risk of AKI in the training set **(A)**, internal validation set **(B),** and external validation set **(C)**. The solid oblique lines represent the 95% confidence interval. AUC, area under the ROC curve.

**FIGURE 4:**
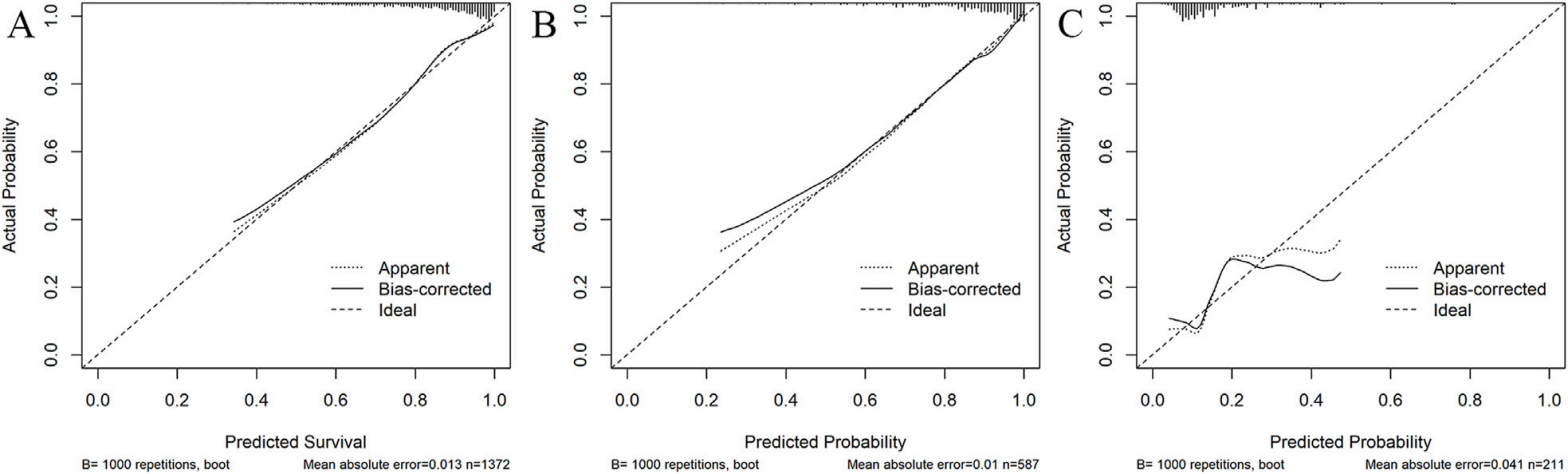
Calibration curves of the predictive nomogram in the training set **(A)**, internal validation set **(B)**, and external validation set **(C)**. The *x*-axis denotes the predicted probability calculated by the nomogram, and the *y*-axis is the observed actual probability of AKI. The diagonal line symbolizes a perfect prediction by an ideal model. The dotted curve illustrates the initial cohort, and the solid curve is bias-corrected by bootstrapping (B = 1,000 repetitions), which shows the bias-corrected performance. The results of the Hosmer–Lemeshow test demonstrate that the *p*-value of the training set **(A)** is 0.255. The *p*-value of the internal validation set **(B)** is 0.357 and that of the external validation set **(C)** is 0.271.

**FIGURE 5:**
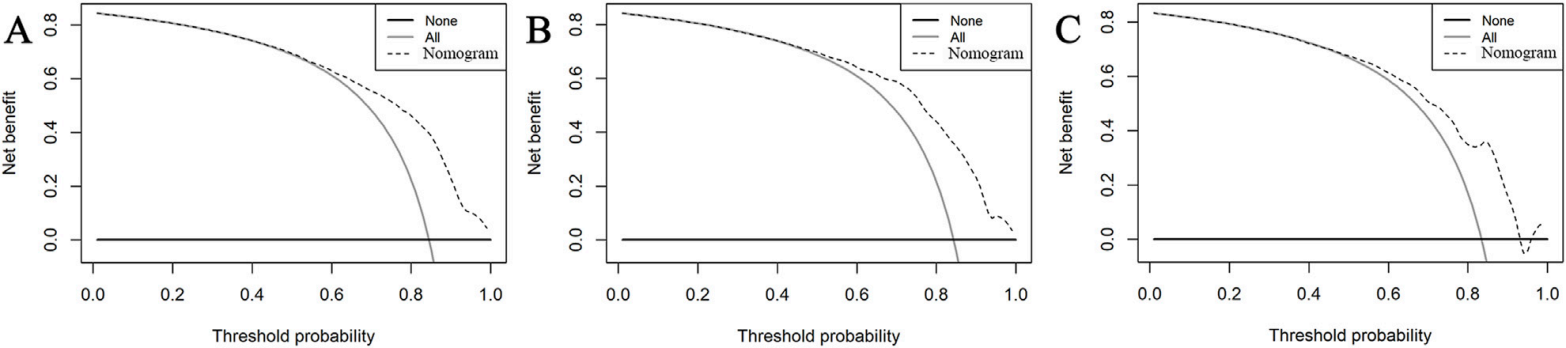
DCA of the nomogram in the training set **(A)**, internal validation set **(B)**, and external validation set **(C)**. In these curves, the horizontal line represents the scenario where no patients develop AKI, while the gray oblique line indicates that all patients develop AKI. The dotted oblique line corresponds to the AKI risk nomogram. The decision curve demonstrates that when the threshold probability of AKI is between 53% and 90%, utilizing this nomogram offers a net benefit over both the treat-all and treat-none strategies.

The AUC of the vancomycin-induced AKI nomogram was 0.791 (95% CI: 0.758–0.823) in the training set, and an AUC of 0.793 (95% CI: 0.742–0.844) was also achieved in the internal validation set. In addition, the AUC value was 0.755 (95% CI: 0.663–0.847) in the external validation set (Figure 3). The calibration plots indicated a close fit for the prediction model in both sets. The H-L test revealed the consistency between the predicted and observed outcomes (training set, χ^2^ = 11.305, *p* = 0.255; internal validation set, χ^2^ = 9.925, *p* = 0.357; and external validation set, χ^2^ = 11.068, *p* = 0.271) (Figure 4). Moreover, the calibration plots also demonstrated good performance (all Brier scores <0.25) (Supplementary Figure S5). The decision curve analysis denoted that the threshold probability for predicting AKI in patients ranged between 53% and 90% (Figure 5), suggesting that using this nomogram for AKI prediction would provide greater clinical benefit than that obtained with standard treat-none or treat-all methods.

### Clinical value comparison between this nomogram and others

To further evaluate the clinical usefulness of the nomogram in this study, we compared NRI and IDI between this nomogram and others. In the training set, we compared this model with the model proposed by Imai. The NRI value was 0.164 (95% CI = 0.090–0.239, *p* < 0.001), while the IDI value was 0.100 (95% CI = 0.080–0.121; *p* < 0.001). In addition, we compared this model with that proposed by Gwak; the NRI value was 0.265 (95% CI = 0.189–0.340; *p* < 0.001), and the IDI value was 0.119 (95% CI = 0.099–0.138; *p* < 0.001) in the training cohort. The above results were confirmed in the data of the validation set (Supplementary Table S2), demonstrating that the nomogram evaluated AKI more accurately than others.

## Discussion

This study centered on AKI following vancomycin administration, aiming to develop a predictive nomogram utilizing routine ICU data to identify risk factors. According to the 2012 KDIGO criteria, our findings revealed that the incidence rate of AKI among ICU patients administered vancomycin is 84.4%. This incidence is marginally higher than the previously published incidence rate (Li et al., 2022), which can be attributed to different inclusion and exclusion criteria, intricate disease profiles, polypharmacy, and various risk factors prevalent in the ICU. Notably, the hospital mortality and the LOS in both the ICU and hospital were significantly elevated in the AKI group compared to the non-AKI group. This disparity underscores the poor prognosis associated with AKI development. Consequently, the proposed nomogram in this study integrates eight predictors: age, weight, vancomycin trough concentration, vancomycin duration, SOFA scores, congestive heart failure, pulmonary diseases, and use of loop diuretics. The validated nomogram is a crucial tool for physicians to detect high-risk patients and evaluate the impact of different risk factors on vancomycin-associated AKI.

Age emerged as a crucial predictor of AKI following vancomycin treatment, with a higher incidence observed in older patients (Li et al., 2022). This increased risk in the elderly may be linked to the increased prevalence of chronic comorbidities, potentially causing more complications during treatment and elevating AKI risk. Additionally, treatment regimens for older patients are often more conservative than those for younger individuals (Coca, 2010). The precise prediction of AKI in elderly patients is crucial for optimizing clinical interventions.

Weight also proved to be a significant predictor of AKI. This study revealed that patients with higher body weight were more prone to AKI. This is likely due to the higher dosages of vancomycin administered to higher-weight patients, correlating with increased AKI risk (Lodise et al., 2008). Interestingly, another study indicated an inverse relationship, where a lower body weight was related to a higher AKI risk (Kim et al., 2022a). This paradox can be attributed to the evaluation of the glomerular filtration rate (eGFR) utilizing the Modification of Diet in Renal Disease (MDRD) formula. In addition, the body mass index (BMI) fails to be calculated due to numerous missing values of height in the MIMIC database. Therefore, the relationship between body weight and kidney injury needs further study.

The vancomycin trough concentration is a crucial indicator. In clinical applications, the loading dose is a problem that must be considered (Katip et al., 2016). A previous study has advocated for maintaining a minimum vancomycin concentration of more than 15 mg/L for treating severe infections to ensure bacterial eradication and improve the patient’s prognosis (Steinmetz et al., 2015). Consistent with this, our findings indicate that increased trough vancomycin concentrations are linked to an increased risk of AKI. Excessively high trough levels of vancomycin may induce AKI and other adverse effects, potentially increasing patient mortality rather than benefiting those with infections. Thus, in clinical practice, it is advisable to aim for lower blood concentrations of vancomycin, provided that effective therapeutic outcomes are assured, to minimize the risk of AKI and provide greater patient benefit. The latest guidelines in 2020 revised the previous treatment recommendations, maintaining vancomycin trough concentrations between 15 and 20 mg/L in severely infected patients to prevent adverse reactions (Rybak et al., 2020b). In addition, it is also an important reference index for predicting renal toxicity. In this study, the results showed that a higher trough concentration of vancomycin was closely associated with a higher incidence of AKI. This finding is consistent with the results from other studies (Wasan et al., 2022). Further studies show that the AUC value calculated according to different trough concentrations can also be used as a more accurate predictor. In non-critical patients, maintaining an area under the curve/minimum inhibitory concentration (AUC/MIC) of ≥400 mg h/L can reduce the incidence of acute kidney injury and maintain better clinical outcomes (Katip and Oberdorfer, 2021). In critically ill patients, the first dose of vancomycin AUC should also be widely used (Sujjavorakul et al., 2023).

The SOFA score was developed to provide an objective quantification of organ dysfunction (Moreno et al., 2023). It has rapidly gained widespread acceptance in adult intensive care, both clinically and in research contexts (Khanna et al., 2017; Hernández et al., 2019). Consistent with our findings, the SOFA score has been identified as a crucial predictor in various models (Toma et al., 2007; Neyra et al., 2023; Lee et al., 2018), underscoring its importance in patient assessment and management.

Cardiac and renal diseases exhibit a complex, bidirectional interplay. Heart failure is often accompanied by various comorbidities, with decreasing renal function being particularly critical (Kellum and Prowle, 2018; Holgado et al., 2020; Chahal et al., 2020). In cases of severe congestive heart failure, as much as 85% of the total blood volume in our body can be redistributed into the veins, leading to arterial underfilling. This arterial hypoperfusion can cause insufficient blood flow to vital organs, especially the kidneys, potentially leading to AKI (Graziani et al., 2014).

Furthermore, a significant interrelationship between kidney and lung functions is evident in both physiological and pathological states. Dysfunction in either organ can directly and indirectly affect another (Bowe et al., 2021). Patients suffering from COPD may present with impaired endothelial function, hypoxemia, elevated activity levels of the sympathetic nerve, and increased aortic stiffness. These factors can contribute to microvascular damage, albuminuria, and deteriorating renal function (Sorino et al., 2019). Moreover, acute lung injury (ALI) and AKI are prevalent in critical patients and are associated with high morbidity and mortality. Exposure to the inflammatory internal environment caused by ALI and injury induced by mechanical ventilation can accelerate the development of AKI (McNicholas et al., 2023; Ahmadian et al., 2021; Darmon et al., 2014).

Loop diuretics are commonly incorporated as a key factor in models predicting vancomycin-related AKI (Xu et al., 2020; Kim et al., 2022b; Miyai et al., 2020; Imai et al., 2017). Although loop diuretics are beneficial for managing edema and hypertension in renal failure patients (Krzych and Czempik, 2019; Ho and Sheridan, 2006), they pose a risk of renal insufficiency. It is thought that loop diuretics may either combine with renal antigens or function as antigens themselves, causing acute interstitial nephritis (Naughton, 2008). Furthermore, they may aggravate renal deterioration by increasing the urine output volume, which may decrease vancomycin distribution and enhance its exposure and toxicity (Hirai et al., 2019). Another study identified the concomitant use of vancomycin and diuretics as an independent risk factor for renal insufficiency among elderly ICU patients (Huang et al., 2018).

Our study has broad implications for clinical administration. First, the nomogram we developed is designed to be user-friendly and easily interpretable by clinicians. These variables incorporated into the nomogram are commonly recorded in ICU settings, which facilitates the practical application of our model without requiring additional tests or complex calculations. The visual representation of the nomogram allows clinicians to quickly assess the risk of vancomycin-associated AKI in individual patients, enabling timely and informed decision-making. In addition, we also performed a decision curve analysis to further evaluate the clinical utility of the model. The results demonstrate that when the threshold probability of AKI is between 53% and 90%, utilizing this nomogram offers a net benefit. Therefore, our nomogram provides significant clinical utility by enabling the early identification of high-risk patients, which is crucial for preventing and managing vancomycin-associated AKI. By accurately predicting the probability of AKI, the model supports clinicians in tailoring treatment plans, adjusting vancomycin dosages, and implementing preventive measures for at-risk patients. This proactive approach can lead to improved patient outcomes, reduced incidence of AKI, and lower healthcare costs associated with prolonged hospital stays and additional treatments.

There are some limitations to this study. First, it is backed by data from a single source for the period 2008–2019, necessitating external validation from diverse medical institutions. Additionally, being retrospective in nature, it lacked some significant predictors. Recent consensus guidelines recommend monitoring the vancomycin efficacy and nephrotoxicity through the AUC/MIC ratio rather than solely relying on trough concentrations (Rybak et al., 2020c). However, due to the limited vancomycin concentration monitoring data, this study could not include this potential factor in the analysis. In addition, due to limitations in the available data of public datasets, we cannot obtain detailed data on the recent biomarkers of kidney injury, such as neutrophil gelatinase-associated lipocalin (NGAL) or kidney injury molecule-1 (KIM-1)(Romejko et al., 2023; Geng et al., 2021). Further studies designed to incorporate these markers are needed to verify their predictive power of vancomycin-associated AKI.

In conclusion, we found that age, weight, vancomycin trough concentration, vancomycin duration, SOFA score, congestive heart failure, pulmonary disease, and use of loop diuretics were predictors of vancomycin-associated AKI in ICU patients. A prediction nomogram for the vancomycin-related AKI was established, and the accuracy of the model was confirmed by internal validation. The personalized prediction model could offer physicians a practical and powerful tool for the early screening and identification of vancomycin-related AKI, which could help guide treatment.

## Data Availability

Publicly available datasets were analyzed in this study. These data can be found at: https://mimic-iv.mit.edu/;MIMIC;ID: 60692864.
