# Supplementary material for: Development and validation of a nomogram to predict the risk of vancomycin-related acute kidney injury in critical care patients
Source: Front Pharmacol. 2024 Aug 28;15:1389140. doi: 10.3389/fphar.2024.1389140 (PMC11387168; doi:10.3389/fphar.2024.1389140)
Supplement: Supplementary file 1 [file DataSheet1.PDF]

frontiers

re-submit supplemental data

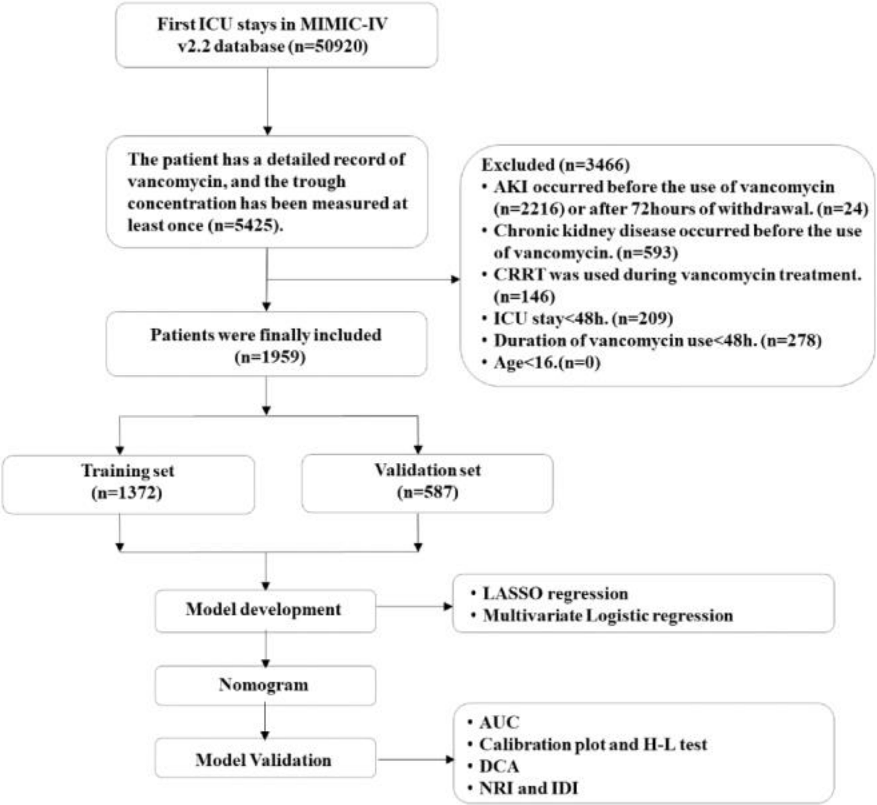

FIGURE S1 The flowchart of the study. ICU intensive care unit, AKI acute kidney injury, LASSO least absolute shrinkage and selection operator, AUC area under the curve, H-L Hosmer-Lemeshow, DCA decision curve analysis. NRI net classification improvement, IDI integrated discrimination improvement.

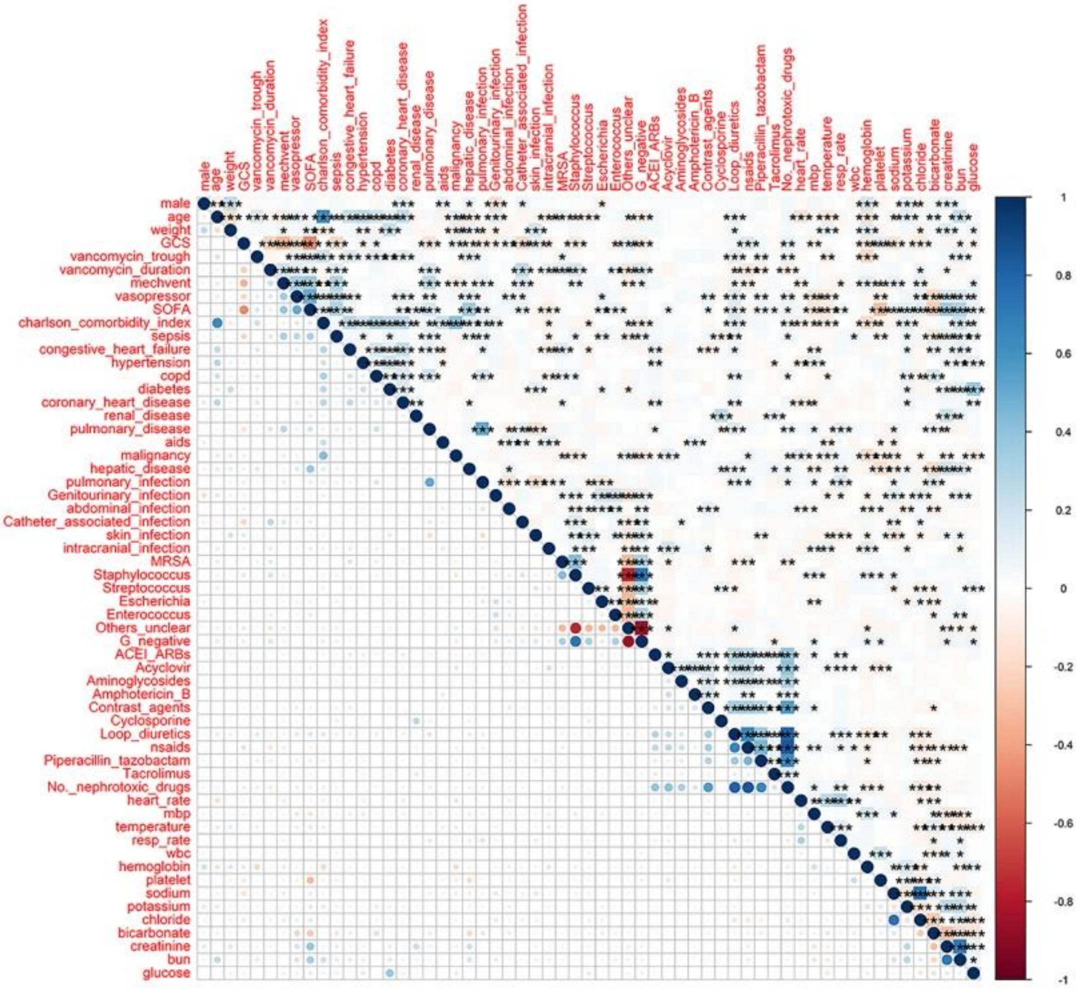

FIGURE S2 The result of correlation analysis among screened variables.

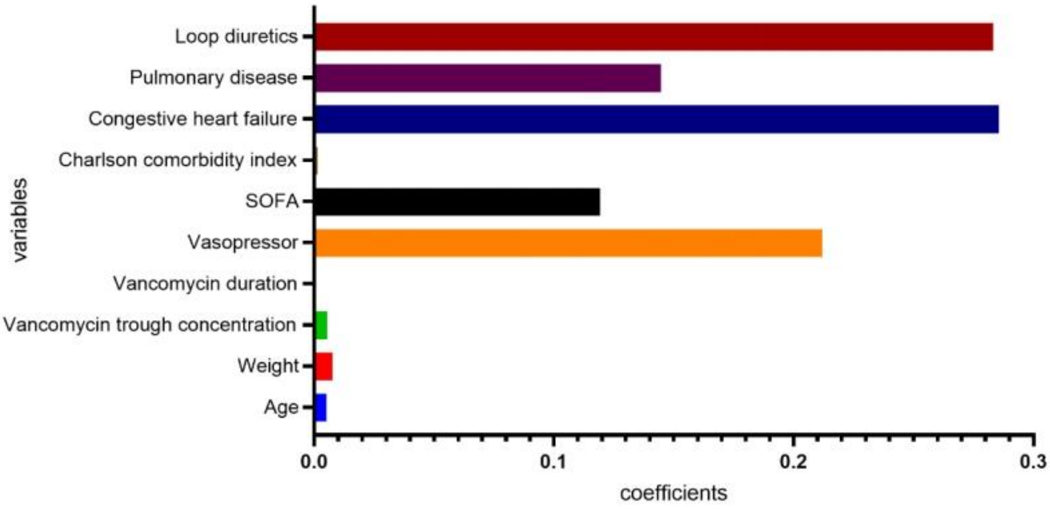

FIGURE S3 The coefficients of 10 potential determinants by LASSO regression.

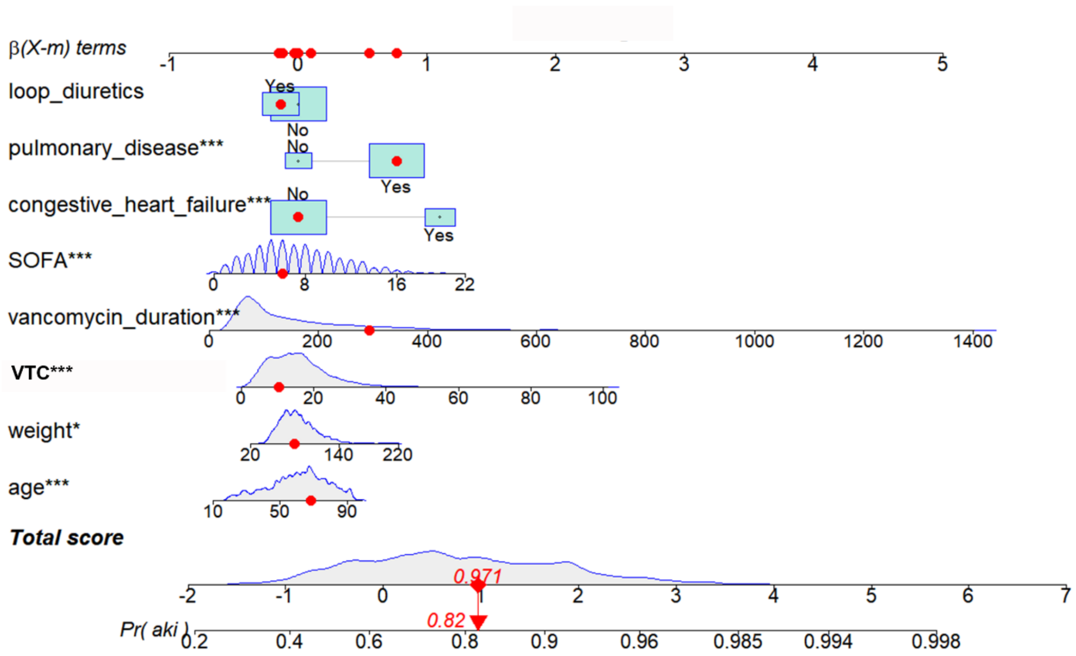

FIGURE S4 Instructions for using a nomogram. The red dot in the line segment corresponding to each variable represents the patient's score for this variable. We add each score in the different variables to get the total score. Finally, we can calculate the probability of vancomycin-associated acute kidney injury according to the total score. Taking the patient as an example, the probability of vancomycin-associated acute kidney injury calculated based on the patient's total score is 82%. VTC, vancomycin through concentration.

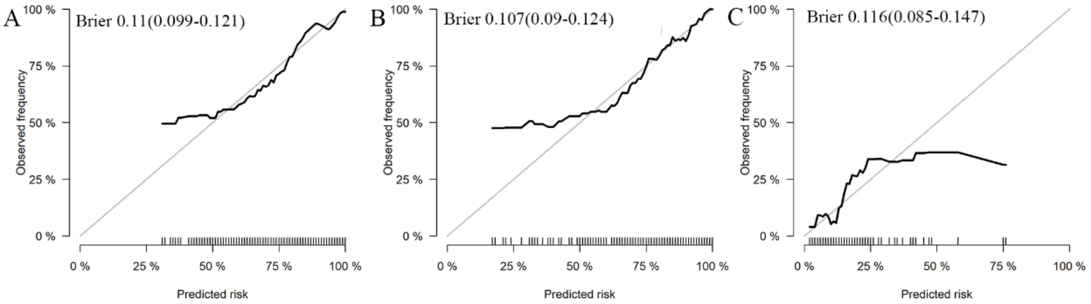

FIGURE S5 Calibration curves of the Nomogram performance by bootstrapping methods (1000 resamples) in estimating vancomycin-associated AKI probability in the training set (A), internal validation set (B), and external validation set (C). Brier score demonstrates goodness of fit. The goodness of fit rises as the Brier score declines.

Table S1 Characteristics of patients in the training and validation sets.

	Training set				Validation set				
	ALL N=1372	Non-AKI N=213	AKI N=1159	*P** value	ALL N=587	Non-AKI N=92	AKI N=495	*P** value	*P*† value
Age(year)	62.8(50.8,74.0)	57.0(46.2,69.5)	63.3(51.8,75.0)	<0.001	63.3(51.4,75.4)	57.0(46.2,69.5)	63.3(51.8,75.0)	0.008	0.774
Gender(male)	769 (56.0%)	112 (52.6%)	657 (56.7%)	0.301	324 (55.2%)	51 (55.4%)	273 (55.2%)	1	0.765
Weight(kg)	80(65.9,97)	71.8(61.2,86.9)	81(67.3,98.9)	<0.001	77.7(65.8,96.1)	71.8(61.3,86.9)	81(67.3,98.9)	<0.001	0.58
GCS	11(7,14)	13(9,14)	11(7,14)	<0.001	11(7,14)	13(9,14)	11(7,14)	<0.001	0.456
SOFA	7(4,10)	4(3,7)	7(5,10)	<0.001	7(4,10)	4(3,7)	7(5,10)	<0.001	0.45
Ventilator	1045 (76.2%)	131 (61.5%)	914 (78.9%)	<0.001	439 (74.8%)	47 (51.1%)	392 (79.2%)	<0.001	0.552
Vasopressor	840 (61.2%)	81 (38.0%)	759 (65.5%)	<0.001	375 (63.9%)	43 (46.7%)	332 (67.1%)	<0.001	0.289
Vancomycin trough concentration (mg/L)	14.8(9.7,20.2)	11.2(7.3,16.5)	15.6(10.4,20.8)	<0.001	15.4(10.5,21.1)	11.2(7.3,16.5)	15.6(10.4,20.8)	0.001	0.165
Vancomycin duration (h)	124(75,223)	92(69.5,170.5)	131(76,235)	<0.001	135(76,244)	92(69.5,170.5)	131(76,235)	<0.001	0.126
**Comorbidities**									
Charlson comorbidity index	5(3,6)	4(2,6)	5(3,6)	<0.001	5(3,7)	4(2,6)	5(3,6)	0.098	0.298
Sepsis	1257 (91.6%)	178 (83.6%)	1079 (93.1%)	<0.001	541 (92.2%)	78 (84.8%)	463 (93.5%)	0.008	0.754
Congestive heart failure	333 (24.3%)	22 (10.3%)	311 (26.8%)	<0.001	138 (23.5%)	14 (15.2%)	124 (25.1%)	0.056	0.761
Hypertension	659 (48.0%)	89 (41.8%)	570 (49.2%)	0.056	282 (48.0%)	32 (34.8%)	250 (50.5%)	0.008	1
COPD	242 (17.6%)	21 (9.86%)	221 (19.1%)	0.002	110 (18.7%)	16 (17.4%)	94 (19.0%)	0.829	0.605
Diabetes	328 (23.9%)	43 (20.2%)	285 (24.6%)	0.195	124 (21.1%)	16 (17.4%)	108 (21.8%)	0.414	0.2
Coronary heart disease	245 (17.9%)	20 (9.39%)	225 (19.4%)	0.001	100 (17.0%)	14 (15.2%)	86 (17.4%)	0.723	0.71
Pulmonary disease	1162 (84.7%)	157 (73.7%)	1005 (86.7%)	<0.001	503 (85.7%)	65 (70.7%)	438 (88.5%)	<0.001	0.62
AIDS	20 (1.46%)	5 (2.35%)	15 (1.29%)	0.221	4 (0.68%)	1 (1.09%)	3 (0.61%)	0.495	0.228
Malignancy:	194 (14.1%)	26 (12.2%)	168 (14.5%)	0.439	90 (15.3%)	16 (17.4%)	74 (14.9%)	0.66	0.538
Hepatic disease	245 (17.9%)	20 (9.39%)	225 (19.4%)	0.001	105 (17.9%)	10 (10.9%)	95 (19.2%)	0.078	1
**Source of infection**									

Pulmonary infection	849 (61.9%)	119 (55.9%)	730 (63.0%)	0.059	384 (65.4%)	50 (54.3%)	334 (67.5%)	0.021	0.152
Genitourinary infection	239 (17.4%)	30 (14.1%)	209 (18.0%)	0.194	113 (19.3%)	13 (14.1%)	100 (20.2%)	0.225	0.367
Abdominal infection	273 (19.9%)	31 (14.6%)	242 (20.9%)	0.042	111 (18.9%)	10 (10.9%)	101 (20.4%)	0.046	0.658
Catheter-associated infection	201 (14.7%)	16 (7.51%)	185 (16.0%)	0.002	84 (14.3%)	11 (12.0%)	73 (14.7%)	0.589	0.9
Skin infection	155 (11.3%)	30 (14.1%)	125 (10.8%)	0.2	53 (9.03%)	12 (13.0%)	41 (8.28%)	0.206	0.158
Intracranial infection	76 (5.54%)	13 (6.10%)	63 (5.44%)	0.819	22 (3.75%)	7 (7.61%)	15 (3.03%)	0.064	0.12
**Infectious pathogen**									
MRSA	112 (8.16%)	12 (5.63%)	100 (8.63%)	0.183	52 (8.86%)	4 (4.35%)	48 (9.70%)	0.145	0.674
Staphylococcus	466 (34.0%)	74 (34.7%)	392 (33.8%)	0.856	205 (34.9%)	24 (26.1%)	181 (36.6%)	0.069	0.721
Streptococcus	121 (8.82%)	30 (14.1%)	91 (7.85%)	0.005	53 (9.03%)	13 (14.1%)	40 (8.08%)	0.097	0.95
Escherichia	111 (8.09%)	18 (8.45%)	93 (8.02%)	0.942	55 (9.37%)	10 (10.9%)	45 (9.09%)	0.732	0.399
Enterococcus	115 (8.38%)	12 (5.63%)	103 (8.89%)	0.15	45 (7.67%)	7 (7.61%)	38 (7.68%)	1	0.66
Others/unclear	719 (52.4%)	112 (52.6%)	607 (52.4%)	1	307 (52.3%)	49 (53.3%)	258 (52.1%)	0.93	1
Co-infection with Gram-negative bacilli	674 (49.1%)	108 (50.7%)	566 (48.8%)	0.669	287 (48.9%)	44 (47.8%)	243 (49.1%)	0.913	0.964
**Concomitant nephrotoxic agent**									
ACEI/ARBs	64 (4.66%)	7 (3.29%)	57 (4.92%)	0.389	13 (2.21%)	1 (1.09%)	12 (2.42%)	0.703	0.015
Acyclovir	69 (5.03%)	10 (4.69%)	59 (5.09%)	0.942	17 (2.90%)	3 (3.26%)	14 (2.83%)	0.739	0.047
Aminoglycosides	34 (2.48%)	5 (2.35%)	29 (2.50%)	1	11 (1.87%)	2 (2.17%)	9 (1.82%)	0.686	0.514
Amphotericin B	5 (0.36%)	1 (0.47%)	4 (0.35%)	0.57	3 (0.51%)	1 (1.09%)	2 (0.40%)	0.401	0.703
Contrast agents	113(8.2%)	10(4.7%)	103(0.09%)	<0.001	42(7.2%)	5(5.4%)	37 (7.5%)	<0.001	0.417
Cyclosporine	1 (0.07%)	0 (0.00%)	1 (0.09%)	1	1 (0.17%)	0 (0.00%)	1 (0.20%)	1	0.51
Loop diuretics	382 (27.8%)	26 (12.2%)	356 (30.7%)	<0.001	144 (24.5%)	18 (19.6%)	126 (25.5%)	0.283	0.145
NSAIDS	564 (41.1%)	76 (35.7%)	488 (42.1%)	0.094	231 (39.4%)	45 (48.9%)	186 (37.6%)	0.054	0.5
Piperacillin tazobactam	225 (16.4%)	23 (10.8%)	202 (17.4%)	0.021	96 (16.4%)	15 (16.3%)	81 (16.4%)	1	1
Tacrolimus	5 (0.36%)	0 (0.00%)	5 (0.43%)	1	5 (0.85%)	2 (2.17%)	3 (0.61%)	0.177	0.177

No. of nephrotoxic drugs	0(0,2)	0(0,2)	0(0,2)	<0.001	0(0,2)	0(0,2)	0(0,2)	0.733	0.11
**Vital signs**									
Heart rate (beats per min)	96(82,111)	97.2(81,111)	96(82,111)	0.584	97(84,110)	97.2(81,111)	96(82,111)	0.208	0.846
MBP (mmHg)	81(70,93)	81(72,95.5)	81(70,93)	0.367	81(70,93)	81(72,95.5)	81(70,93)	0.215	0.264
Temperature(◦C)	36.9(36.5,37.4)	37.1(36.6,37.5)	36.9(36.5,37.4)	0.358	36.9(36.5,37.3)	37.1(36.6,37.5)	36.9(36.5,37.4)	0.102	0.444
Respiratory rate (breaths per min)	21(17,25)	20(16.5,24)	21(17,25.5)	0.017	20(16,25)	20(16.5,24)	21(17,25.5)	0.488	0.237
Laboratory tests									
WBC(K/uL)	13(8.9,18.3)	12.8(8.3,17.1)	13.1(19,18.5)	0.501	12.6(8.6,18.3)	12.8(8.3,17.2)	13.1(9,18.5)	0.538	0.415
Hemoglobin(g/dl)	11(9.4,12.9)	11.3(9.3,12.8)	11(9.4,12.9)	0.432	11.1(9.4,13)	11.3(9.3,12.8)	11(9.4,12.9)	0.045	0.969
Platelet(K/uL)	219.5(148.3,304)	239(14.7,305.5)	217(149,304)	0.362	210(142,308)	239(147.5,305.5)	217(149,304)	0.696	0.588
Sodium(mmol/L)	138(135,141)	138.0(136,141)	138(135,141)	0.522	138(135,141)	138(136,141)	138(135,141)	0.081	0.421
Potassium(mmol/L)	4.1(3.7,4.5)	3.9(3.5,4.3)	4.1(3.7,4.6)	<0.001	4.1(3.6,4.6)	3.9(3.5,4.3)	4.1(3.7,4.6)	0.927	0.529
Chloride(mmol/L)	103(99,107)	103(99,106.5)	103(99,107)	0.595	103(99,108)	103(99,106.5)	103(99,107)	0.061	0.35
Bicarbonate(mmol/L)	22(20,25)	23(20,25.5)	22(19,25)	0.143	23(20,26)	23(20,25.5)	22(19,25)	0.833	0.092
Creatinine(mg/dl)	0.9(0.7,1.2)	0.8(0.6,1.2)	0.9(0.7,1.2)	0.959	0.9(0.7,1.3)	0.8(0.6,1.2)	0.9(0.7,1.2)	0.076	0.074
BUN (mg/dl)	18(13,29)	15(11,23.5)	19(13,30)	0.097	18(13,29)	15(11,23.5)	19(13,30)	0.091	0.582
Glucose(mg/dl)	132(108,147.8)	127(109.5,161.5)	133(107,177)	0.805	131(109,174)	127(109.5,161.5)	133(107,177)	0.065	0.33
**Outcomes**									
LOS ICU (days)	7.1(4.1,12.8)	4.3(2.9,7.4)	7.7(4.7,13.3)	<0.001	6.7(4.2,12.2)	4.3(2.9,7.4)	7.7(4.7,13.3)	<0.001	0.841
LOS hospital(days)	14.6(9.2,22.9)	12.2(7.3,18.9)	15.1(9.8,23.3)	<0.001	14.3(8.8,22.7)	12.2(7.3,18.9)	15.1(9.8,23.3)	0.003	0.76
Hospital mortality	246 (17.9%)	10 (4.69%)	236 (20.4%)	<0.001	111 (18.9%)	10 (10.9%)	101 (20.4%)	0.046	0.652

Characteristics are summarized as median (Q1, Q3) or frequency (%). Abbreviations: AKI Acute kidney injury, GCS Glasgow coma scale, SOFA Sequential organ failure assessment, COPD Chronic obstructive pulmonary disease, AIDS Acquired immunodeficiency syndrome, MRSA Methicillin-resistant Staphylococcus aureus, ACEI/ARBs angiotensin-converting enzyme inhibitors/Angiotensin receptor blockers, NSAIDs Non-steroidal anti-inflammatory drugs, MBP Mean blood pressure, WBC White blood cell, BUN Blood urea nitrogen, LOS Length of stay. **p* value for difference between patients with AKI versus non AKI. †*p* value for training set versus validation set for overall characteristics.

Table S2 Comparison of different models.

		NRI			IDI		
		Estimate	95%CI	*P* value	Estimate	95%CI	*P* value
this model vs Imai model	Training set	0.164	0.090 - 0.239	< 0.001	0.100	0.080 - 0.121	< 0.001
	Validation set	0.210	0.089 - 0.331	< 0.001	0.171	0.133 - 0.209	< 0.001
this model vs Gwak model	Training set	0.265	0.189 - 0.340	< 0.001	0.119	0.099 - 0.138	< 0.001
	Validation set	0.195	0.088 - 0.302	< 0.001	0.159	0.120 - 0.198	< 0.001

NRI net reclassification improvement, IDI integrated differentiation improvement.
